# Supplementary material for: Knot Formation on DNA Pushed Inside Chiral Nanochannels
Source: Polymers (Basel). 2023 Oct 22;15(20):4185. doi: 10.3390/polym15204185 (PMC10611388; doi:10.3390/polym15204185)
Supplement: Supplementary file 1 [file polymers-15-04185-s001.zip › polymers-2612646-supplementary.pdf]

polymers

Supplementary Information

Knot formation on DNA pushed inside chiral nanochannels

Renáta Rusková and Dušan Račko

Polymer Institute of the Slovak Academy of Sciences, Dúbravská cesta 9, 845 41 Bratislava, Slovak; renata.ruskova@savba.sk (R.R.); dusan.racko@savba.sk (D.R.)

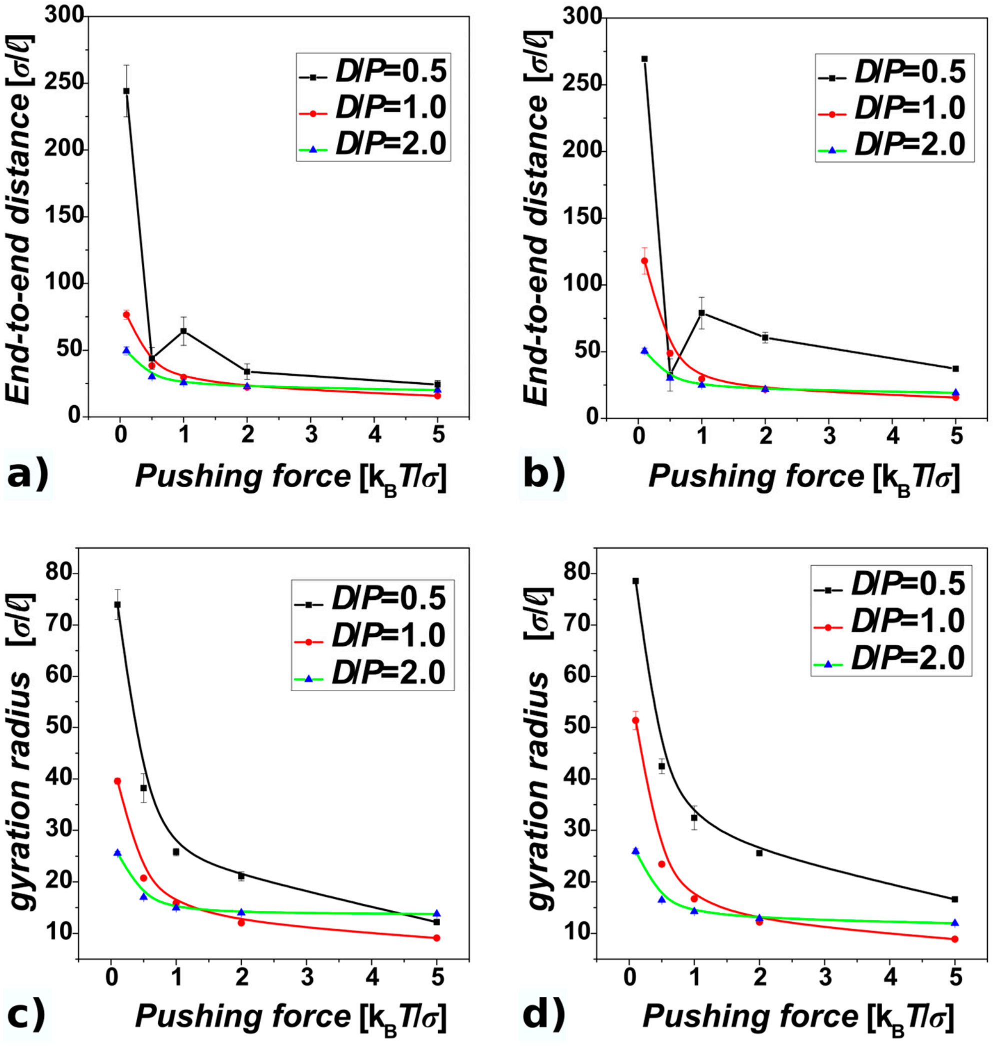

Figure S1. Polymer metrics as a function of pushing force in confinements with different strengths. Panels a) and b) compare end-to-end distance computed on polymers pushed inside a) cylindrical and b) helical channels with forces F = 0.1, 0.5, 1, 2 and 5 k_BT/σ. The end-to-end distance is shown in units of σ/ℓ, where σ is the size of the bead and ℓ is the equilibrium bond length. The end-to-end distance is obtained as an average over 50,000 structures. The end-to-end distance drops with increasing pushing force. The graphs show sudden drop of the end-to-end distance at F = 0.5 k_BT/σ, which corresponds to backfolded regime when the polymer conformations remain in U-shaped hairpin conformation. Panels at the bottom row show gyration radii computed in c) cylindrical and d) helical channels for the same force and confinement strengths as used to computed end-to-end distances in panels a) and b). The graphs for D/P=0.5 and 1.0 show a steady decrease of gyration as the chain compactifies by the increasing pushing force. In the case of D/P=2.0, the

gyration radius at the highest force slightly increases, what is a result of intense spooling of the polymer chains, and polymer chain effects when there is not enough monomers to effectively fill the largest channel.

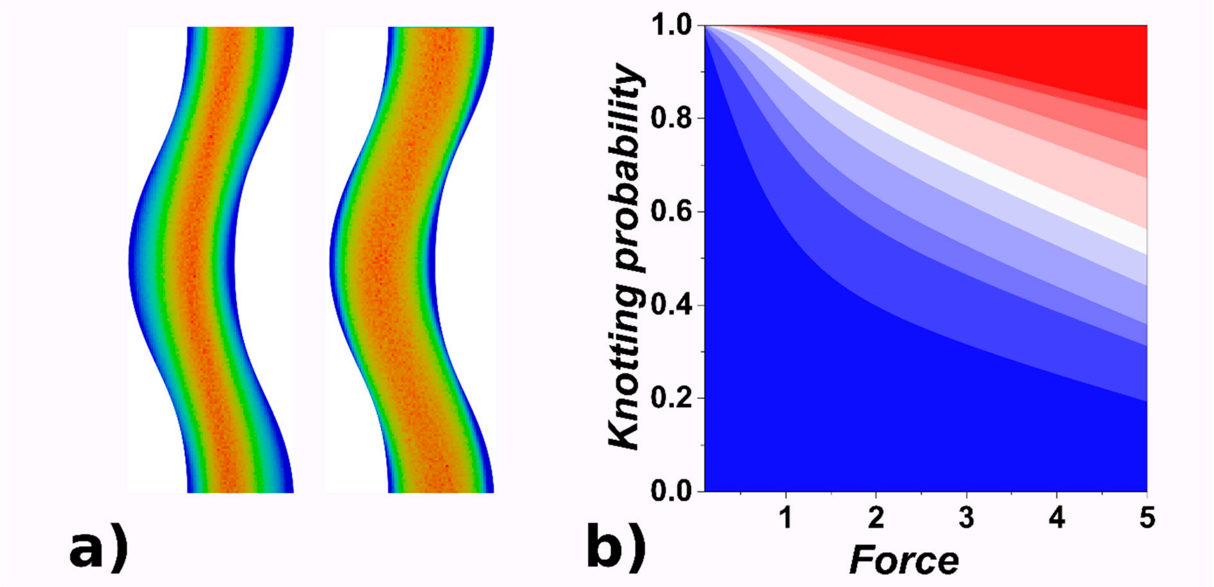

Figure S2. The monomer distributions and knotting probabilities in helical channel with the pitch of the channel set to 2 P. The confinement strength of the channels in terms of D/P ratio is 0.5. a) The panel shows planar projections of monomer distributions for smallest and largest velocities of pushing from the range of settings used in the simulations. b) The panel shows knotting probabilities as the function of pushing force, i.e. velocities (see the inset of Figure 1a in the main text). The colours represent populations of knots with increasing complexity in terms of crossing number as stacked areas.

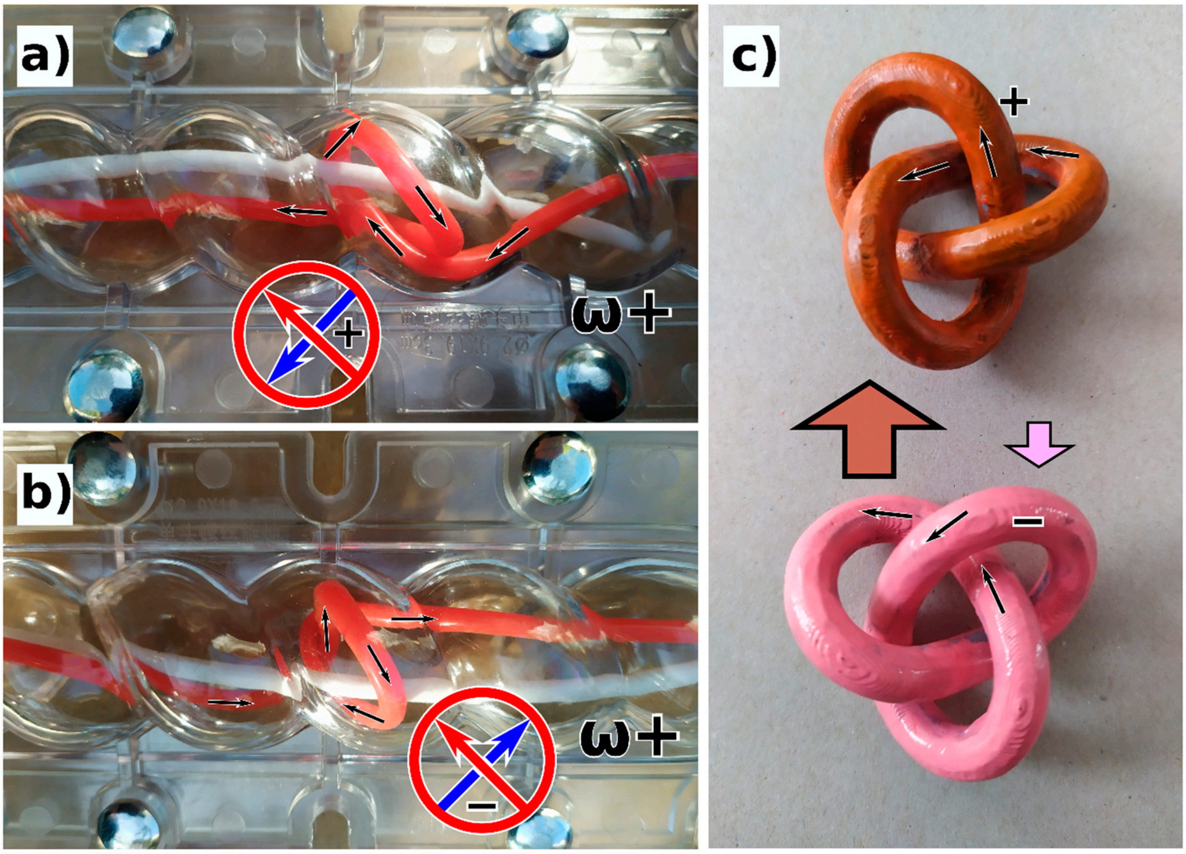

Figure S3. Physical analogue model is used as a demonstrator of how the helical channels induce handedness to knots. a) and b) It is built from a rubber tube showing DNA, transparent helical mould representing righthanded nanochannel and c) 3D printed righthanded and lefthanded knots. The demonstrator shows, that in order to produce a knot by the mechanism of looping and threading (white tube shows the threaded portion) a loop with at least one crossing has to be first created by twisting of a portion of the polymer, that is then threaded by the DNA strand. b) In order to fit the loop with opposite handedness than is the winding of the channel, the loop has to be twisted by a half-turn more than in the case of equichiral loops (as shown on the panel a). This may cause producing of antichiral knots is less likely than the equichiral knots.

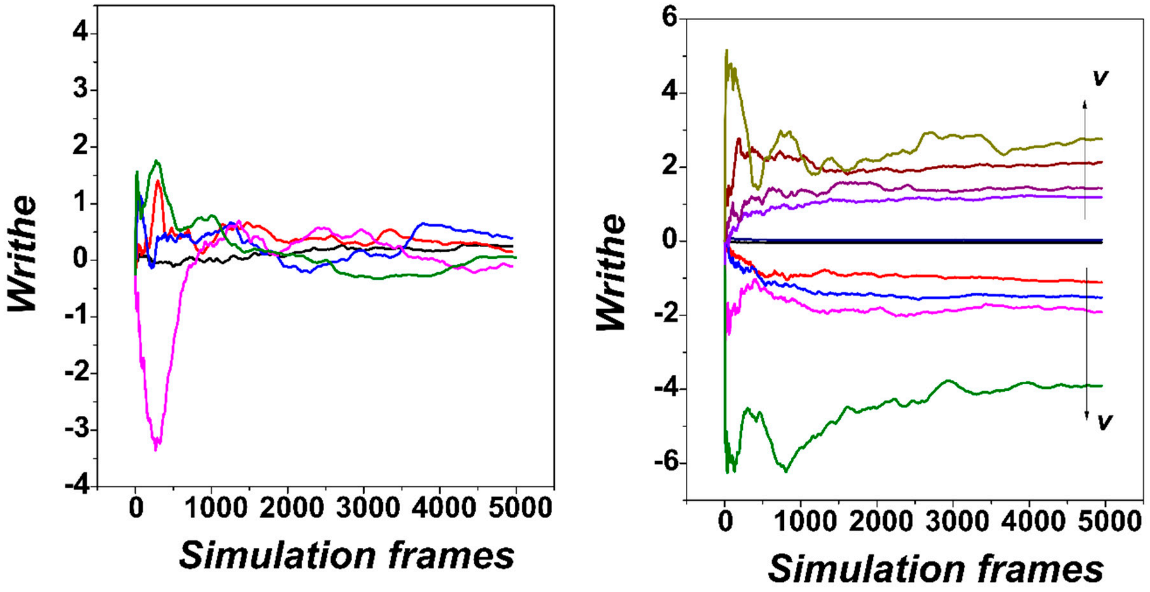

Figure S4. Evolution of writhe with the simulation time. The simulation time is represented in terms of consecutive frames that were analysed. The picture to the left shows the evolution of writhe in cylindrical channels. The picture to the left shows the evolution of writhe in helical channels, where the lines in the bottom half of the figure were computed for channels with negative winding of the helical loops, ω-, and the lines in the top half of the picture were obtained in righthanded channels, ω+. Confinement strength is indicated in terms of v increasing in the direction of the arrow.
